# The association between the dietary inflammatory index and allergic rhinitis: a case–control study

**DOI:** 10.3389/fnut.2024.1418305

**Published:** 2024-06-27

**Authors:** Qian Wang, Niuniu Dong, Yan Feng, Yan Ning, Ruifang Zhu, Shifan Han

**Affiliations:** ^1^School of Nursing, Shanxi Medical University, Taiyuan, China; ^2^Editorial Department, First Hospital of Shanxi Medical University, Taiyuan, China

**Keywords:** allergic rhinitis, dietary inflammatory index (DII), diet, inflammation, case–control study

## Abstract

**Background and objective:**

Allergic rhinitis (AR) is a common chronic inflammatory disease that significantly impacts the quality of life of patients. However, there is limited research on the relationship between the Dietary Inflammatory Index (DII) and the risk of AR. Our study aimed to assess the association between DII and AR in a sample of adults from North China.

**Methods:**

In a case–control study, we selected 166 cases of AR and 166 age- and gender-matched controls. Dietary intake was assessed using a validated food frequency questionnaire. The energy-adjusted DII (E-DII) scores were calculated based on the quantity of diet components with inflammatory or anti-inflammatory potential. We used conditional logistic regression models to examine the association between E-DII and AR.

**Results:**

Our findings indicate a positive correlation between E-DII and AR risk. After controlling for confounders, individuals in the highest E-DII tertile exhibited a 4.41-fold increased risk of AR compared to those in the lowest tertile (OR 4.41, 95% CI 2.31–8.41). Additionally, stratified analysis showed that E-DII was positively associated with AR subtype (seasonal vs. perennial), duration (≤6 years vs. >6 years), severity (mild vs. moderate–severe), and onset time (intermittent vs. persistent). Furthermore, individuals in the highest E-DII tertile had higher intake of total fat, SFA, PUFAs, and n-6 PUFAs.

**Conclusion:**

In conclusion, we realized that there is a positive association between the E-DII score and AR. The consumption of diets abundant in anti-inflammatory nutrients and low in pro-inflammatory nutrient contents is recommended as a preventative strategy against AR.

## 1 Introduction

Allergic Rhinitis (AR) is a common chronic inflammatory condition characterized by Immunoglobulin E (IgE)-mediated hypersensitivity to various allergens (1). The typical clinical features of AR include nasal itching, sneezing, congestion, and rhinorrhea, along with prevalent ocular symptoms such as red eyes and itching. These symptoms not only disrupt daily activities, learning, and sleep for affected individuals but also have a significant impact on their overall quality of life. AR poses a global health concern, affecting an estimated 20–40% of the world’s population. In the United States, self-reported AR prevalence ranges from 10 to 30% in adults and up to 40% in children. In Canada, the prevalence of physician-diagnosed AR is as high as 20% (2). In China, the prevalence of AR is on the rise. The self-reported prevalence of AR was 8.3% in Guangzhou, southern China (3). The self-reported prevalence of AR in Beijing, northern China, was 19.1% (4). Despite the continuous advancement of therapeutic approaches, including the widespread application of immunotherapy, the prevalence of AR remains high.

The etiology of AR is multifactorial, involving a complex interplay of genetic predispositions, environmental factors, age, lifestyle habits, and geographical location, with intricate pathophysiological mechanisms (5). The pathogenesis of AR primarily involves an aberrant immune response to environmental allergens, leading to the hyperactivation of Th2 cell-mediated immune responses. These cells produce key cytokines such as IL-4, IL-5, and IL-13, which promote the production of IgE and the activation of eosinophils. Additionally, this process triggers the release of histamine and other mediators from inflammatory cells, including mast cells and basophils, resulting in inflammatory reactions in the nasal mucosa, characterized by congestion, edema, and allergic symptoms (6). This inflammatory process is further modulated by various factors, including lifestyle-related risk factors, that can either exacerbate or mitigate the inflammatory response through its impact on the body’s inflammatory profile.

Considering the significant role of lifestyle factors in modulating inflammatory responses, it is essential to investigate how modifiable risk factors such as unhealthy dietary practices, lack of physical activity, obesity, and chronic stress can exacerbate the progression of AR (7, 8). Among these modifiable factors, diet emerges as a significant adjustable element, offering a potential avenue for the prevention and management of AR through lifestyle modifications. Emerging research indicates that dietary modifications can play a significant role in mitigating the risk of allergic diseases (9–11). A randomized controlled study found that an increased intake of fruits and vegetables was associated with observed improvements in airway reactivity among children in the intervention group. This suggests that a diet rich in fruits and vegetables may have potential benefits for childhood asthma through antioxidant components and anti-inflammatory pathways (12). A Mendelian randomization study exploring the causal relationship between fatty acids and atopic dermatitis (AD) found that higher levels of omega-3 fatty acids (n-3PUFAs) were associated with a reduced risk of AD. This suggests that n-3PUFAs may confer a protective effect (13).

The relationship between diet and inflammation has garnered widespread attention. The Dietary Inflammatory Index (DII) has emerged as a valuable instrument for evaluating the influence of diet on systemic inflammation (14). Validation of the DII is evidenced by its association with six key inflammatory markers, including Interleukin-4 (IL-4), IL-10, IL-6, IL-1β, Tumor Necrosis Factor-alpha (TNF-α), and C-reactive Protein (CRP). Among them, pro-inflammatory cytokines include: IL-1β, IL-6, TNF-α, and CRP, anti-inflammatory cytokines include: IL-4 or IL-10. The modulation of inflammation by food parameters is manifested through three discernible conditions: a pro-inflammatory effect denoted by an increase in IL-1β, IL-6, TNF-α, or CRP levels, or a decrease in IL-4 or IL-10; an anti-inflammatory effect indicated by a reduction in IL-1β, IL-6, TNF-α, or CRP levels, or an increase in IL-4 or IL-10; and a condition where food parameters do not induce any notable alterations in inflammatory markers (15). The DII assigns a score ranging from +1 to −1 to individual foods based on their impact on inflammatory markers. Positive scores suggest a potential to increase inflammation, while negative scores indicate a potential to decrease inflammation. A higher DII score reflects a diet that may intensify an ongoing inflammatory response, whereas a lower DII score is linked to a diet that may help mitigate inflammation.

The DII has emerged as a valuable tool in nutritional epidemiology, with applications in numerous studies examining links between diet and disease risks, such as cancer (16), asthma (17), cardiovascular and cerebrovascular diseases (18), type 2 diabetes (19), and osteoporosis (20). While these studies have affirmed the DII’s role in assessing dietary inflammatory potential, its application in AR remains largely unexplored. Addressing this gap, our case–control study among the Chinese population introduces a novel perspective. By examining the relationship between DII and AR risk, our research potentially offers insights into the prevention and management strategies for AR. Our study aims to provide original data that can enrich the understanding of diet-disease connections in the context of AR.

## 2 Materials and methods

### 2.1 Participants

This case–control study was carried out at the outpatient clinic of the First Hospital of Shanxi Medical University between July and October 2023, involving a total of 332 participants matched in a 1:1 ratio. Inclusion criteria for the case group were as follows: (1) meeting the diagnostic criteria for AR according to the Chinese Guidelines for the Diagnosis and Treatment of Allergic Rhinitis (2022, revised edition) (1); (2) age ≥ 18 years. The control group was sourced from hospital visitors, health check-up centers, and outpatients without an AR diagnosis, with the control population also being ≥18 years of age. Cases and controls were matched 1:1 based on gender and age, with age matching done by age groups (e.g., 18–20, 21–25, 26–30, and so on). Exclusion criteria for both groups included: (1) coexistence of any disease that could potentially affect dietary habits, including hypertension, diabetes, hyperlipidemia, cardiovascular diseases, gout, functional gastrointestinal disorders, fatty liver, malignant tumors, and other severe gastrointestinal diseases; (2) being pregnant or breastfeeding; (3) recent changes in dietary habits.

### 2.2 Assessment of dietary intake

Dietary information was obtained through the administration of a validated semi-quantitative Chinese Food Frequency Questionnaire (FFQ), which evaluated food consumption over the previous 3 months (21, 22). The FFQ encompassed 16 major food categories, including staple foods, meats and meat products, eggs, seafood, legumes and legume products, vegetables, pickled vegetables, mushrooms, snacks and nuts, fruits, dairy products, alcoholic beverages, non-alcoholic beverages, cooking oils, condiments, and dietary supplements, totaling 106 food items.

Participants were instructed to report the frequency of consumption and average portion size of a variety of foods during the designated survey period, with frequency levels categorized into eight categories (≥3 times/day, 2 times/day, 1 time/day, 5–6 times/week, 3–4 times/week, 1–2 times/week, 1–3 times/month, <1 time/month). To aid participants in quantifying their intake, researchers provided images depicting different portion sizes of the foods in question.

Based on the FFQ data, participants’ food consumption data was transformed into daily intake quantities. Daily dietary nutrient intakes were calculated using the data from the Chinese Food Composition Table (23). Participants with incomplete FFQ responses or extreme energy intake levels (less than 500 kcal or greater than 4,000 kcal per day) were excluded from the statistical analysis.

### 2.3 Assessment of E-DII scores

In the current study, we calculated DII scores using the validated methodology proposed by Shivappa et al. (15). This approach was developed after an extensive literature review from 1950 to 2010 that examined the dietary influence on six inflammatory markers: IL-1β, IL-4, IL-6, IL-10, TNF-α, and CRP. The comprehensive review identified 45 food parameters associated with changes in these markers. Each parameter was given an inflammatory effect score from −1 to 1, reflecting the collective evidence from the number of studies, their design, and the nature of their findings—anti-inflammatory, pro-inflammatory, or neutral. A negative score indicates an anti-inflammatory effect, 0 indicates no significant effect, and a positive score suggests a pro-inflammatory effect, with higher absolute values representing stronger effects.

In order to reduce variability stemming from individual initial intake values, the intake of each food parameter was standardized using the global average intake and standard deviation from 11 countries, resulting in Z-scores. These Z-scores were then adjusted to centered proportions to mitigate the effects of right skewness. The individual’s centered proportion for each food parameter was multiplied by its corresponding food parameter effect score, based on the inflammatory potential of each food parameter as determined by the literature review, to calculate the individual’s food parameter-specific DII score (15, 24). The sum of all food parameter-specific DII scores was used to generate an overall DII score for each participant in the study. Energy-adjusted DII (E-DII) scores were then computed using the density method, which involved converting all food parameters to a standardized unit of 1,000 kcal of nutrients (25). In this study, the available components for calculating E-DII included the following 28 nutrients: energy, carbohydrates, dietary fiber, protein, total fat, cholesterol, saturated fatty acids (SFA), polyunsaturated fatty acids (PUFAs), monounsaturated fatty acids (MUFAs), n-3PUFAs, omega-6 fatty acids (n-6PUFAs), β-carotene, isoflavones, folate, niacin, riboflavin, thiamine, vitamin A, vitamin C, vitamin D, vitamin E, vitamin B12, vitamin B6; magnesium, Fe, Zn, Se, and alcohol.

### 2.4 Assessment of covariates

Professionally trained researchers conducted in-person interviews to gather fundamental clinical data, encompassing demographic details such as age, gender, marital status, education level, occupation, smoking and alcohol consumption history, physical activity, and body mass index (BMI), with BMI computed as weight in kilograms divided by the square of height in meters. Furthermore, for the case group, additional information was obtained pertaining to the type, duration, and severity of the disease at the time of definitive diagnosis.

### 2.5 Ethics statement

This study adhered to the guidelines of the Helsinki Declaration and was approved by the Ethical Committee of the First Hospital of Shanxi Medical University (IRB Ref. No.: KYLL-2024-087). All participants signed an informed consent form to participate in the research. Data collection followed the reporting guidelines of the Strengthening the Reporting of Observational Studies in Epidemiology (STROBE) statement for observational studies (26).

### 2.6 Statistical analysis

Continuous variables are presented as mean ± standard deviation, and categorical variables are presented as number (percentage). The normality of continuous variables was evaluated through the Kolmogorov–Smirnov test. Differences between continuous variables were compared using Student’s *t*-test (ANOVA) or Mann–Whitney test (Kruskal-Wallis test), and differences between categorical variables were assessed using the chi-square test. Participants were stratified into tertiles of E-DII distribution within the control group, with the E-DII groups denoted as T1, T2, and T3, respectively, with T1 serving as the reference group. Logistic regression analyses were conducted to determine the odds ratios (OR) and 95% confidence intervals (95% CI) for the relationship between E-DII and AR risk. Covariates adjusted for in the logistic regression models encompassed gender, age, education level, occupation, smoking status, alcohol consumption, physical activity, and BMI. In the regression models, the median of each group was used as a continuous variable for linear trend testing. Stratified analyses of E-DII and AR risk were conducted according to the type, duration, severity, timing of onset, and allergen types of AR. All statistical analyses were carried out using SPSS version 25.0, with a significance level of *p* < 0.05 considered statistically significant.

## 3 Results

This study included a cohort of 166 patients diagnosed with AR and 166 age- and gender-matched control subjects. Analysis revealed that AR patients exhibited significantly higher E-DII scores compared to the control group (*p* < 0.001), with the control group scoring an average of −0.91 ± 1.44 and the AR group scoring 0.36 ± 1.82. Demographic variables such as age, gender, BMI, education level, and daily activity were well-matched between the two groups, with no statistically significant differences observed, except for occupation, which also showed significant variation between the groups (see Table 1). The E-DII scores ranged from −3.62 to 4.94 for AR patients and from −3.96 to 3.58 for controls. Notably, the distribution of E-DII scores within the AR group indicated a tendency toward pro-inflammatory dietary patterns, as evidenced by the statistically significant difference in comparison to the control group. The categorization of E-DII scores into tertiles (T1: ≤ −1.48, T2: between −1.48 and − 0.36, T3: > −0.36) further illustrates the dietary inflammatory potential, with T1 reflecting anti-inflammatory diets and T3 indicating pro-inflammatory diets. The median dietary inflammatory potential is represented by T2, which falls between the thresholds of T1 and T3.

**Table 1:** Overall study participant characteristics.

Variables		Mean ± SD or N (%)		*p*-value
		Cases (*n* = 166)	Controls (*n* = 166)	
Body mass index (BMI, kg/m^2^)^a^		22.28 ± 3.03	22.15 ± 3.49	0.350
Total energy (kcal)^a^		2412.78 ± 670.62	2600.73 ± 734.23	0.028
E-DII^a^		(−0.91) ± 1.44	0.36 ± 1.82	<0.001
Age (years)^a^		32.07 ± 10.30	31.62 ± 9.60	0.856
Sex^b^	Males	73(44.0%)	73(44.0%)	1.000
	Females	93(56.0%)	93(56.0%)	
Education^b^	Junior middle school	31(18.7%)	28(16.9%)	0.586
	Senior middle school	32(19.3%)	29(17.5%)	
	Junior college	30(18.1%)	36(21.7%)	
	Bachelor’s degree	53(31.9%)	45(27.1%)	
	Graduate degree	20(12.0%)	28(16.9%)	
Occupation^b^	Employee/Student	110(66.3%)	75(45.2%)	<0.001
	Retired	3(1.8%)	5(3.0%)	
	Unemployed	13(7.8%)	41(24.7%)	
	Freelancer	40(24.1%)	45(27.1%)	
Smoking^b^	Yes	37(22.3%)	45(27.1%)	0.309
	No	129(77.7%)	121(72.9%)	
Alcohol^b^	Yes	21(12.7%)	30(18.1%)	0.334
	No	145(87.3%)	136(81.9%)	
Physical activity^b^	Low activity	98(59.0%)	90(54.2%)	0.665
	Moderate activity	43(25.9%)	47(28.3%)	
	Severe activity	25(15.1%)	29(17.5%)	

BMI, body mass index; E-DII, energy-adjusted dietary inflammatory index. ^a^*p*-values were obtained from independent Student’s *t*-tests or c2 tests, where appropriate. ^b^Data are numbers (%), and were analyzed by chi-squared test or Fisher’s exact test.

The sociodemographic characteristics of the study population, categorized by E-DII tertiles, are detailed in Table 2. Significant disparities were observed in the educational attainment levels among the control group (*p* = 0.003), with a notably lower percentage of individuals with a college education or below in groups T2 and T3 compared to group T1. Conversely, a higher proportion of individuals with a bachelor’s degree or higher was observed in group T1. Among patients with AR, there were no significant differences in age, gender, education level, occupation, smoking status, primary activities, or BMI distribution among groups T1, T2, and T3. These findings suggest that, within the AR patient cohort, the distribution of E-DII scores does not appear to be associated with the sociodemographic variables assessed.

**Table 2:** Distribution of food groups across tertiles of E-DII.

Variables			E-DII			*P*-value
			T1 < (−1.48)	T2 (−1.48 to −0.36)	T3 > (−0.36)	
BMI (kg/m^2^)^a^		Cases	22.32 ± 2.92	22.17 ± 2.99	22.34 ± 3.23	0.942
		Controls	22.58 ± 2.22	22.83 ± 4.22	22.81 ± 3.68	0.937
Total energy (kcal)^a^		Cases	2284.49 ± 632.86	2498.69 ± 583.19	2458.24 ± 771.25	0.130
		Controls	2692.82 ± 658.25	2574.92 ± 715.36	2582.54 ± 767.51	0.674
Age (years)^a^		Cases	32.98 ± 10.53	32.70 ± 11.18	30.55 ± 9.12	0.455
		Controls	32.57 ± 10.49	32.37 ± 8.64	31.04 ± 9.73	0.573
Sex^b^	Males	Cases	25(44.6%)	21(38.9%)	27(48.2%)	0.611
	Females	Cases	31(55.4%)	33(61.1%)	29(51.8%)	
	Males	Controls	12(40.0%)	14(36.8%)	47(48.0%)	1.609
	Females	Controls	18(60.0%)	24(63.2%)	51(52.0%)	
Education^b^	Junior middle school	Cases	16(28.6%)	7(13.0%)	5(8.9%)	0.003
	Senior middle school	Cases	11(19.6%)	12(22.2%)	9(16.1%)	
	Junior college	Cases	15(26.8%)	10(18.5%)	5(8.9%)	
	Bachelor’s degree	Cases	9(16.1%)	18(33.3%)	27(48.2%)	
	Graduate degree	Cases	5(8.9%)	7(13.0%)	10(17.9%)	
	Junior middle school	Controls	9(30.0%)	6(15.8%)	13(13.3%)	0.095
	Senior middle school	Controls	6(20.0%)	7(18.4%)	16(16.3%)	
	Junior college	Controls	6(20.0%)	10(26.3%)	20(20.4%)	
	Bachelor’s degree	Controls	2(6.7%)	8(21.1%)	35(35.7%)	
	Graduate degree	Controls	7(23.3%)	7(18.4%)	14(14.3%)	
Occupation^b^	Employee/Student	Cases	31(55.4%)	37(68.5%)	42(75.0%)	0.359
	Retired	Cases	2(3.6%)	0(0%)	1(1.8%)	
	Unemployed	Cases	6(10.7%)	4(7.4%)	3(5.4%)	
	Freelancer	Cases	17(30.4%)	13(24.1%)	10(17.9%)	
	Employee/Student	Controls	10(33.3%)	17(44.7%)	48(49.0%)	0.655
	Retired	Controls	1(3.3%)	2(5.3%)	2(2.0%)	
	Unemployed	Controls	8(26.7%)	10(26.3%)	23(23.5%)	
	Freelancer	Controls	11(36.7%)	9(23.7%)	25(25.5%)	
Smoking^b^	Yes	Cases	8(14.3%)	13(24.1%)	16(28.6%)	0.179
	No	Cases	48(85.7%)	41(75.9%)	40(71.4%)	
	Yes	Controls	7(23.3%)	10(26.3%)	28(28.6%)	0.846
	No	Controls	23(76.7%)	28(73.7%)	70(71.4%)	
Alcohol^b^	Yes	Cases	5(8.9%)	6(11.1%)	10(17.9%)	0.334
	No	Cases	51(91.1%)	48(88.9%)	46(82.1%)	
	Yes	Controls	6(20.0%)	8(21.1%)	16(16.3%)	0.777
	No	Controls	24(80.0%)	30(78.9%)	82(83.7%)	
Physical activity^b^	Low activity	Cases	35(62.5%)	32(59.3%)	31(55.4%)	0.916
	Moderate activity	Cases	13(23.2%)	15(27.8%)	15(26.8%)	
	Severe activity	Cases	8(14.3%)	7(13.0%)	10(17.9%)	
	Low activity	Controls	13(43.3%)	20(52.6%)	57(58.2%)	0.697
	Moderate activity	Controls	10(33.3%)	11(28.9%)	26(26.5%)	
	Severe activity	Controls	7(23.3%)	7(18.4%)	15(15.3%)	

BMI, body mass index; E-DII, energy-adjusted dietary inflammatory index. ^a^The One-way analysis of variance (ANOVA) was used for comparison of the variables between E-DII tristiles. ^b^Data are numbers (%), and were analyzed by chi-squared test or Fisher’s exact test.

The daily dietary nutrient intake of the study participants is detailed in Table 3, revealing that the case group exhibited higher consumption of total fat, SFA, MUFAs, and PUFAs compared to the control group (*p* < 0.05 for each nutrient). Conversely, the intake of total protein, carbohydrates, fiber, cholesterol, n-3PUFAs, vitamin A, vitamin B6, vitamin C, vitamin E, magnesium (Mg), iron (Fe), zinc (Zn), selenium (Se), riboflavin, niacin, folate, β-carotene, and isoflavones was lower in the case group (*p* < 0.05 for each nutrient). Within the case group, higher E-DII scores were linked to higher intake of total fat, SFA, PUFAs, and n-6 PUFAs, as well as lower intakes of protein, carbohydrates, fiber, vitamin C, vitamin E, Mg, Fe, Zn, folate, β-carotene, isoflavones, n-3 PUFAs, vitamin B6, riboflavin, thiamine, and vitamin A (*p* < 0.05 for each comparison). No significant differences were observed in the intake of cholesterol, niacin, Se, MUFAs, alcohol, vitamin B12, and vitamin D in the case group with increased E-DII scores. Suggesting that these nutrients are not associated with the risk of AR in our study population.

**Table 3:** Dietary intakes of the study participants included in E-DII (energy per 1,000 kcal).

	Cases (*n* = 166)	Controls (*n* = 166)	*P*-value^a^	E-DII (cases)^b^			*P*-value^b^
	Mean ± SD	Mean ± SD		T1(<−1.48)	T2(−1.48 to −0.36)	T3(> − 0.36)	
Proteins (g)	30.94 ± 6.87	37.00 ± 6.09	<0.001	33.27 ± 6.70	33.62 ± 7.35	29.19 ± 6.22	0.003
Total fats (g)	58.99 ± 10.33	51.56 ± 9.84	<0.001	54.46 ± 8.36	56.79 ± 9.67	61.24 ± 10.57	0.002
Carbohydrates (g)	86.23 ± 21.50	99.52 ± 20.29	<0.001	96.89 ± 16.10	91.38 ± 19.41	80.97 ± 22.17	<0.001
Dietary fiber (g)	8.24 ± 3.94	10.56 ± 3.42	<0.001	13.48 ± 4.26	10.43 ± 1.27	5.78 ± 1.96	<0.001
Cholesterol (mg)	271.75 ± 152.26	325.93 ± 195.20	0.006	290.96 ± 190.16	279.93 ± 138.95	262.69 ± 145.04	0.535
Thiamine (mg)	0.35 ± 0.08	0.34 ± 0.06	0.354	0.37 ± 0.08	0.37 ± 0.07	0.33 ± 0.07	0.033
Riboflavin (mg)	0.34 ± 0.08	0.40 ± 0.10	<0.001	0.37 ± 0.07	0.38 ± 0.10	0.32 ± 0.07	0.001
Niacin (mg)	5.36 ± 1.60	5.89 ± 1.73	0.002	5.3 ± 1.23	5.47 ± 1.99	5.33 ± 1.55	0.993
Vitamin A (μg RE)	257.16 ± 161.45	314.73 ± 163.81	<0.001	465.08 ± 234.90	293.90 ± 87.19	179.27 ± 70.11	<0.001
Vitamin B6(mg)	0.30 ± 0.13	0.36 ± 0.14	<0.001	0.44 ± 0.16	0.34 ± 0.10	0.23 ± 0.09	<0.001
Vitamin B12(μg)	0.05 ± 0.05	0.04 ± 0.05	0.496	0.04 ± 0.03	0.06 ± 0.07	0.04 ± 0.04	0.602
Vitamin C (mg)	50.17 ± 43.06	67.12 ± 41.80	<0.001	108.76 ± 61.25	60.00 ± 19.01	28.42 ± 17.81	<0.001
Vitamin D (μg)	135.83 ± 146.31	132.38 ± 138.97	0.551	112.9 ± 87.03	167.68 ± 224.85	130.50 ± 119.49	0.541
Vitamin E (mg)	10.93 ± 5.44	15.48 ± 4.79	<0.001	17.44 ± 5.77	13.77 ± 2.36	7.85 ± 3.51	<0.001
Mg (mg)	138.74 ± 37.29	174.93 ± 34.29	<0.001	172.01 ± 32.28	160.55 ± 26.66	120.10 ± 30.06	<0.001
Fe (mg)	9.47 ± 1.67	11.05 ± 2.07	<0.001	10.18 ± 1.65	10.33 ± 1.66	8.92 ± 1.45	<0.001
Zn (mg)	4.46 ± 0.86	5.48 ± 0.90	<0.001	4.95 ± 0.80	5.04 ± 0.84	4.08 ± 0.67	<0.001
Se (μg)	19.98 ± 5.52	24.28 ± 5.79	<0.001	20.79 ± 4.59	21.16 ± 6.33	19.28 ± 5.39	0.140
SFA (g)	12.20 ± 3.06	10.32 ± 2.65	<0.001	11.07 ± 2.87	11.42 ± 2.67	12.85 ± 3.12	0.002
MUFAs (g)	22.31 ± 5.15	19.83 ± 5.60	<0.001	20.67 ± 5.15	22.37 ± 5.26	22.79 ± 5.05	0.089
PUFAs (g)	12.13 ± 3.92	9.62 ± 2.58	<0.001	10.67 ± 3.22	11.10 ± 3.73	12.98 ± 4.00	0.012
n-3PUFAs (g)	0.74 ± 0.63	1.12 ± 0.87	<0.001	0.9 ± 0.81	1.15 ± 0.76	0.53 ± 0.36	<0.001
n-6PUFAs (g)	18.19 ± 5.63	18.10 ± 6.36	0.532	15.62 ± 5.77	18.66 ± 6.14	20.11 ± 6.44	<0.001
Folate (μg)	49.44 ± 21.47	66.69 ± 25.57	<0.001	71.63 ± 23.07	60.07 ± 15.21	38.52 ± 14.83	<0.001
b-carotene (mg)	1298.22 ± 1230.08	1689.43 ± 1220.91	<0.001	2975.03 ± 1801.77	1526.00 ± 548.26	696.60 ± 482.68	<0.001
Isoflavone (mg)	4.20 ± 3.70	6.51 ± 5.14	<0.001	5.61 ± 3.77	5.17 ± 3.48	3.38 ± 3.57	<0.001
Alcohol (g)	2.39 ± 6.90	1.44 ± 2.79	0.524	1.79 ± 3.89	1.40 ± 2.69	2.97 ± 8.54	0.801

SFA, saturated fatty acid; MUFA, monounsaturated fatty acids; PUFA, polyunsaturated fatty acids. ^a^*P*-values were obtained from independent Student’s *t*-tests or c2 tests, where appropriate. ^b^The One-way analysis of variance (ANOVA) was used for comparison of the variables between DII quartiles.

The OR for the associations between both continuous and categorical E-DII and the risk of AR are presented in Table 4. In the univariate analysis, an increment in E-DII scores, treated as a continuous variable, was significantly associated with an elevated risk of AR (OR 1.68, 95% CI 1.42–1.99, *p* < 0.001). This significant association was consistent in the multivariable-adjusted model, indicating that for each unit increase in E-DII score, there is a 68% increase in the odds of AR, after controlling for confounding factors (OR 1.68, 95% CI 1.42–1.99, *p* < 0.001). When E-DII scores were examined categorically, the group with the highest E-DII scores had a markedly higher risk of AR compared to the group with the lowest scores, with an odds ratio of 3.27 (95% CI 1.88–5.67, *p* < 0.001). This elevated risk was further pronounced after adjusting for potential confounders, with an adjusted odds ratio of 4.41 (95% CI 2.31–8.41, *p* < 0.001), underscoring the robustness of the association between a pro-inflammatory diet and the risk of AR. These findings underscore the importance of dietary inflammatory potential in the etiology of AR.

**Table 4:** OR and 95% CI for association between the E-DII and the risk of AR.

	E-DII continuous	*P*-value	Tertiles of E-DII			*P*-value
	OR (95%CI)		T1(<−1.48)	T2(−1.48 to −0.36)	T3(> − 0.36)	
				OR (95%CI)	OR (95%CI)	
N: control/case			30/56	38/54	98/56	
Cases/Controls	1.60(1.38–1.85)	<0.001	1	1.31(0.716–2.41)	3.27(1.88–5.67)	<0.001
Multivariate-adjuste^a^ d ^a^	1.68(1.42–1.99)	<0.001	1	1.70(0.84–3.44)	4.41(2.31–8.41)	<0.001

E-DII, energy-adjusted dietary inflammatory index; OR, odds ratio; CI, confidence intervals. ^a^Fully adjusted for age, sex, education, occupation, smoking, Alcohol, physical activity and body mass index.

Subsequently, we performed stratified analyses to examine the association between E-DII and AR risk based on various factors such as AR type, duration, severity, and timing of onset. The results of these analyses are presented in Table 5. Our findings indicate a positive correlation between E-DII and the risk of AR across all evaluated categories. Specifically, this association was observed regardless of whether the AR was seasonal or perennial, the duration was ≤6 years or > 6 years, the severity was mild or moderate–severe, and the onset was intermittent or persistent. The odds of developing AR were significantly higher with each increment in E-DII score within each stratum (*p* < 0.05 for all comparisons). These findings suggest that a dietary pattern characterized by a higher E-DII score, indicative of a pro-inflammatory diet, is associated with an elevated risk of AR. Importantly, this association appears to be consistent across different clinical presentations of AR, highlighting the potential impact of diet on the development and possibly the progression of the condition.

**Table 5:** Stratified analysis of E-DII versus AR risk.

Variables		E-DII			*P*-value
		T1(<−1.48)	T2(−1.48 to −0.36)	T3(> − 0.36)	
			OR (95%CI)	OR (95%CI)	
Type of AR					
Seasonal	N: control/case	20/56	23/54	61/56	
	Cases/Controls	1	1.19(0.59–2.42)	3.05(1.63–5.71)	<0.001
	Multivariate-adjusted^a^	1	1.34(0.59–3.07)	5.15(2.29–11.58)	<0.001
Perennial	N: control/case	9/56	15/54	38/56	
	Cases/Controls	1	1.73(0.70–4.28)	4.22(1.87–9.54)	<0.001
	Multivariate-adjusted^a^	1	1.82(0.59–5.64)	9.49(3.26–27.64)	<0.001
Disease duration					
≤6 years	N: control/case	18/56	25/54	64/56	
	Cases/Controls	1	1.44(0.71–2.94)	3.56(1.87–6.75)	<0.001
	Multivariate-adjusted^a^	1	1.78(0.75–4.23)	6.18(2.67–14.31)	<0.001
>6 years	N: control/case	11/56	13/54	35/56	
	Cases/Controls	1	1.23(0.51–2.97)	3.18(1.47–6.89)	0.001
	Multivariate-adjusted^a^	1	1.16(0.42–3.25)	5.80(2.17–15.53)	0.001
Disease severity					
Mild	N: control/case	13/56	17/54	40/56	
	Cases/Controls	1	1.36(0.60–3.06)	3.08(1.49–6.37)	0.001
	Multivariate-adjusted^a^	1	1.45(0.55–3.81)	4.99(2.01–12.35)	0.001
Medium and severe	N: control/case	16/56	21/54	59/56	
	Cases/Controls	1	1.36(0.64–2.88)	3.69(1.90–7.17)	<0.001
	Multivariate-adjusted^a^	1	1.68(0.68–4.12)	7.50(3.09–18.25)	<0.001
Symptom onset time					
Intermittent	N: control/case	11/56	16/54	48/56	
	Cases/Controls	1	1.51(0.64–3.54)	4.36(2.06–9.26)	<0.001
	Multivariate-adjusted^a^	1	1.89(0.69–5.22)	8.95(3.36–23.87)	<0.001
Persistent	N: control/case	18/56	22/54	51/56	
	Cases/Controls	1	1.27(0.61–2.62)	2.83(1.48–5.44)	0.001
	Multivariate-adjusted^a^	1	1.70(0.71–4.06)	4.92(2.14–11.31)	0.001

E-DII, energy-adjusted dietary inflammatory index; OR, odds ratio; CI, confidence intervals. ^a^Fully adjusted for age, sex, education, occupation, smoking, Alcohol, physical activity and body mass index.

## 4 Discussion

To our knowledge, this is the first study to specifically investigate the relationship between the E-DII and the risk of adult AR. While previous research has explored the association between the DII and other allergic conditions, such as asthma and AD (27, 28), research on AR remains scarce. In this case–control study, we identified a significant positive correlation between the E-DII and the risk of AR. Subsequent stratified analyses indicated a positive relationship between E-DII and various clinical manifestations of AR, including seasonal and perennial patterns, duration of illness, severity, and onset times. This association remained significant even after adjusting for potential confounders such as age, gender, BMI, and other risk factors. Furthermore, individuals with higher E-DII scores demonstrated a substantially elevated risk of AR compared to those with lower DII scores, with a risk ratio reaching 4.41. This finding aligns with the results of a cross-sectional study conducted in South Korea on pediatric AR, which also indicates a positive correlation between a high DII score and an increased risk of AR, with a risk ratio reaching 2.23 (29). Moreover, we observed that individuals in the highest E-DII tertile tended to consume higher amounts of pro-inflammatory nutrients, including total fat, SFA, and n-6PUFAs, while consuming lower amounts of anti-inflammatory nutrients such as dietary fiber, vitamins, trace elements, and phytochemicals. This discovery suggests a plausible biological pathway through which an imbalance in the consumption of pro-inflammatory and anti-inflammatory nutrients may lead to the development of an inflammatory environment, potentially heightening the risk of AR.

Diet may play a role in the development and progression of AR through its inflammatory potential. Previous studies have explored the impact of specific dietary components on AR. This study further elucidates this relationship: we found that high intakes of total fat, SFA, PUFAs, and n-6 PUFAs can increase the risk of AR, whereas high intakes of protein, carbohydrates, dietary fiber, vitamin A, riboflavin, vitamin C, vitamin E, magnesium, Fe, Zn, folate, beta-carotene, isoflavones, n-3 PUFAs, vitamin B6, and thiamine are associated with a reduced risk of AR. These results are consistent with the findings of the majority of existing studies. Multiple studies have also indicated that a high-fat diet may increase the risk of developing AR (30, 31). Notably, our study’s findings reveal that in the case group, the intake of MUFAs and PUFAs is higher than in the control group. Further analysis found that individuals in the case group within the highest tertile of the E-DII consume higher amounts of PUFAs and n-6 PUFAs, and tend to have lower intakes of n-3 PUFAs. It is well recognized that unsaturated fatty acids have a multitude of positive impacts on health, including aiding in weight control, promoting stable blood sugar levels, and possessing anti-inflammatory and immune-modulating capabilities (32). Unsaturated fatty acids primarily consist of MUFAs and PUFAs. PUFAs encompass both n-3 PUFAs and n-6 PUFAs. n-3 PUFAs are primarily derived from deep-sea fish and certain plant seeds, and their anti-inflammatory role in AR has been confirmed by a substantial amount of animal experiments and human studies (33–35). On the other hand, while n-6 PUFAs play a significant role in cellular signal transduction and modulation of inflammatory responses, excessive intake may exacerbate inflammatory reactions by promoting the production of certain inflammatory mediators (36), potentially worsening the symptoms of AR to some extent. These findings provide a clear elucidation of our results. Current research indicates that nutrients such as vitamins A, D, and E, along with minerals like zinc, iron, and selenium, possess not only antioxidant properties but also anti-inflammatory effects, aiding in immune system regulation and inflammation mitigation (10). Vitamin A supports epithelial cell integrity, while vitamins D and E can reduce inflammatory biomarkers (37). Minerals like zinc, iron, and selenium are crucial for modulating the activity of various enzymes involved in controlling oxidative stress and inflammation (38, 39). Li et al. study suggests that vitamin D supplementation may reduce the risk of asthma exacerbation in children with serum 25(OH)D levels below 10 ng/mL and alleviate symptoms of AD and AR, potentially through mechanisms involving the suppression of Th2 cell function and reduction of IgE secretion (40). Jiang et al. found that vitamin E, especially combined with selenium, can reduce AR and asthma symptoms by potentially modulating immunity, as well as antioxidant and anti-inflammatory effects (41). Dietary fiber positively affects host immune responses by promoting gut health and balancing the gut microbiota, which is closely related to anti-inflammatory processes. A Korean cross-sectional study showed that higher intake of dietary fiber is associated with a reduced prevalence of asthma, allergic rhinitis, and AD in adults. Dietary fiber may regulate immune responses and affect allergic diseases by increasing gut microbial diversity and the production of short-chain fatty acids (42). Phytochemicals such as flavonoids and polyphenols, abundant in fruits, vegetables, and whole grains, have anti-inflammatory and immunomodulatory effects, inhibiting inflammatory pathways and reducing the production of inflammatory cytokines. Clinical trials in Iran found that pomegranate supplements can improve clinical symptoms in allergic asthma patients and decrease counts of eosinophils, basophils, and neutrophils, likely through its antioxidant and anti-inflammatory properties (43). These findings suggest that modifying dietary structures and supplementing with specific vitamins, trace elements, and phytochemicals may aid in preventing or mitigating the risk of AR. Further studies could delve into the impact of particular nutrients and dietary patterns on AR to devise more targeted interventions.

Beyond specific dietary components, the dietary pattern has been shown to be associated with AR. Previous studies have indicated that adherence to the Mediterranean diet (MedDiet) is associated with a decreased risk of asthma and AR, a finding that aligns with the primary focus of our study. The MedDiet, recognized as an anti-inflammatory dietary pattern, is characterized by a high intake of vegetables, legumes, fruits, nuts, whole grains, seafood, and olive oil, with moderate wine consumption and reduced intake of meat and dairy products. This dietary habit is believed to have a lower pro-inflammatory potential, attributed to its rich content of anti-inflammatory components and limited presence of pro-inflammatory elements (44). For instance, a cross-sectional study in Mexico indicated that adherence to the MedDiet pattern is correlated with a lower risk of AR (OR = 0.41; 95% CI = 0.22–0.77). Similarly, a cross-sectional study conducted in Crete also found that a high adherence to the MedDiet offers protective effects against AR (OR = 0.34; 95% CI = 0.18–0.64) (45). These study findings corroborate our observations that an anti-inflammatory diet may play a positive role in preventing AR. Conversely, a study conducted in Turkey found no significant relationship between adherence to the MedDiet and the risk of developing AR (46). The discrepancies may arise from variations in study design, sample selection, and regional dietary patterns. In summary, the MedDiet, known for its anti-inflammatory effects, has been linked to a decrease in the DII score. A randomized controlled trial conducted among older Australians showed that adherence to the MedDiet for 6 months significantly reduced DII scores (47). This decrease in DII scores implies a potential reduction in inflammation, which could have a positive impact on AR.

The pathogenesis of AR is closely associated with inflammatory responses (48). The DII, which assesses the relative content of pro- and anti-inflammatory nutrients in an individual’s dietary habits, may indirectly influence the pathogenesis of AR. Specifically, high DII scores are typically associated with higher intakes of pro-inflammatory nutrients, such as SFA, and lower intakes of anti-inflammatory nutrients, such as n-3PUFAs and dietary fiber. This imbalance may lead to an overproduction of pro-inflammatory cytokines in the body, such as IL-6, IL-1β, and TNF-α (15). These cytokines promote Th2 type immune responses and the activation of inflammatory cells, exacerbating the symptoms of rhinitis. Concurrently, DII may also influence the production of anti-inflammatory cytokines such as IL-10, which possess suppressive effects on inflammation. A reduction in IL-10 levels may decrease the inhibition of inflammatory responses, thereby potentially exacerbating the development of AR (49, 50). Furthermore, DII could further promote inflammatory processes by affecting the levels of CRP, an acute phase protein whose increased levels are typically associated with inflammatory states (51). Thus, DII may indirectly modulate the pathophysiological processes of AR by regulating the expression and activity of these key inflammatory markers.

This study has several strengths. Firstly, it is a meticulously designed case–control study that reveals, for the first time, a potential link between the DII and the risk of AR. Secondly, the case and control groups were matched for age and gender and recruited from the same medical center. Thirdly, our analysis took into account multiple potential confounding variables, thereby reducing bias. Fourthly, we used a validated semi-quantitative Chinese food frequency questionnaire (FFQ) to collect dietary data from participants, which covers the main foods in the Chinese population’s diet. However, there are also some limitations to our study. The FFQ may have measurement errors, as participants may overestimate or underestimate the intake of certain foods intentionally or unintentionally when recalling their diet, requiring accurate assessment by experienced professionals. Due to limitations of the FFQ and the Chinese food composition table, our E-DII calculation included only 28 dietary nutrients, with another 17 food parameters not included in the current data, which may affect our results. Additionally, the relatively small sample size and the restriction to the population in Shanxi Province, China, may limit the generalizability of our findings to a broader population. Future research should be conducted with larger samples and in diverse regional populations to validate our findings and further explore the relationship between DII and the risk of AR. Furthermore, while our study focused on examining the influence of the DII on the risk of AR, we did not specifically investigate individual food allergens. However, recognizing their potential role in the pathogenesis of AR, we suggest that future research should explore the interactions between food allergens, DII, and other dietary factors more thoroughly.

## 5 Conclusion

In summary, in this case–control study, we recognized a positive correlation between E-DII scores and the risk of AR. Additionally, E-DII scores were associated with various clinical manifestations of AR. These findings suggest that a pro-inflammatory diet may exacerbate the development and manifestations of AR by enhancing systemic inflammatory responses. This discovery contributes to the field while emphasizing the potential of dietary interventions as a preventive strategy for AR. Future research should aim to validate these findings through large-scale, prospective cohort studies to establish a robust association between dietary inflammatory properties and AR risk. It is also essential to explore the impact of cultural and regional dietary habits on AR risk, as this could inform the development of targeted public health strategies and personalized dietary recommendations for individuals at risk of AR. By adopting a comprehensive approach that considers individual, cultural, and regional factors, future studies can enhance our understanding of the intricate relationship between diet and AR. This knowledge can ultimately guide the formulation of effective preventive measures and personalized dietary interventions, improving the quality of life for those affected by AR.

## Data availability statement

The raw data supporting the conclusions of this article will be made available by the authors, without undue reservation.

## Ethics statement

The studies involving humans were approved by the Ethical Committee of the First Hospital of Shanxi Medical University. The studies were conducted in accordance with the local legislation and institutional requirements. The participants provided their written informed consent to participate in this study.

## Author contributions

QW: Data curation, Investigation, Methodology, Writing – original draft. ND: Data curation, Investigation, Writing – review & editing. YF: Methodology, Resources, Writing – review & editing. YN: Data curation, Software, Writing – review & editing. RZ: Conceptualization, Methodology, Project administration, Writing – review & editing. SH: Conceptualization, Methodology, Project administration, Writing – review & editing.

## References

[1] Subspecialty Group of Rhinology, Editorial Board of Chinese Journal of Otorhinolaryngology Head and Neck Surgery, Subspecialty Group of Rhinology, Society of Otorhinolaryngology Head and Neck Surgery, Chinese Medical Association. Chinese guideline for diagnosis and treatment of allergic rhinitis. Zhonghua Er Bi Yan Hou Tou Jing Wai Ke Za Zhi. (2022) 57:106–29. doi: 10.3760/cma.j.cn115330-20211228-00828 35078291

[2] Dykewicz MS Wallace DV Amrol DJ Baroody FM Bernstein JA Craig TJ. Rhinitis 2020: a practice parameter update. J Allergy Clin Immunol. (2020) 146:721–67. doi: 10.1016/j.jaci.2020.07.007 32707227

[3] Li CW Chen DH Zhong JT Lin ZB Peng H Lu HG. Epidemiological characterization and risk factors of allergic rhinitis in the general population in Guangzhou City in China. PLoS One. (2014) 9:e114950. doi: 10.1371/journal.pone.0114950 25514026 PMC4267734

[4] Zheng M Wang X Bo M Wang K Zhao Y He F. Prevalence of allergic rhinitis among adults in urban and rural areas of China: a population-based cross-sectional survey. Allergy Asthma Immunol Res. (2015) 7:148. doi: 10.4168/aair.2015.7.2.148 25729622 PMC4341336

[5] Meng Y Wang C Zhang L. Recent developments and highlights in allergic rhinitis. Allergy. (2019) 74:2320–8. doi: 10.1111/all.14067 31571226

[6] Zhang Y Lan F Zhang L. Update on pathomechanisms and treatments in allergic rhinitis. Allergy. (2022) 77:3309–19. doi: 10.1111/all.15454 35892225

[7] Zhang Y Lan F Zhang L. Advances and highlights in allergic rhinitis. Allergy. (2021) 76:3383–9. doi: 10.1111/all.15044 34379805

[8] Park J-H Yoo E Seo M-W Jung HC Lee J-M. Association between physical activity and respiratory diseases in adolescents: an age- and gender-matched study. Int J Environ Res Public Health. (2021) 18:1397. doi: 10.3390/ijerph18041397 33546335 PMC7913582

[9] Venter C Eyerich S Sarin T Klatt KC. Nutrition and the immune system: a complicated tango. Nutrients. (2020) 12:818. doi: 10.3390/nu12030818 32204518 PMC7146186

[10] Zhang P. The role of diet and nutrition in allergic diseases. Nutrients. (2023) 15:3683. doi: 10.3390/nu15173683 37686715 PMC10490368

[11] Nobs SP Zmora N Elinav E. Nutrition regulates innate immunity in health and disease. Annu Rev Nutr. (2020) 40:189–219. doi: 10.1146/annurev-nutr-120919-094440 32520640

[12] Berthon BS McLoughlin RF Jensen ME Hosseini B Williams EJ Baines KJ. The effects of increasing fruit and vegetable intake in children with asthma: a randomized controlled trial. Clin Exp Allergy. (2021) 51:1144–56. doi: 10.1111/cea.13979 34197676

[13] Lin J-Y Ma L-J Yuan J-P Yu P Bai B-X. Causal effects of fatty acids on atopic dermatitis: a Mendelian randomization study. Front Nutr. (2023) 10:1083455. doi: 10.3389/fnut.2023.1083455 36908902 PMC9996175

[14] Phillips CM Chen L-W Heude B Bernard JY Harvey NC Duijts L. Dietary inflammatory index and non-communicable disease risk: a narrative review. Nutrients. (2019) 11:1873. doi: 10.3390/nu11081873 31408965 PMC6722630

[15] Shivappa N Steck SE Hurley TG Hussey JR Hébert JR. Designing and developing a literature-derived, population-based dietary inflammatory index. Public Health Nutr. (2014) 17:1689–96. doi: 10.1017/S1368980013002115 23941862 PMC3925198

[16] Wu J Yu C Shivappa N Hébert JR Xu X. Dietary inflammatory index and renal cancer risk: a prospective study. Food Funct. (2023) 14:9287–94. doi: 10.1039/D3FO02158K 37779467

[17] Visser E De Jong K Van Zutphen T Kerstjens HAM Ten Brinke A. Dietary inflammatory index and clinical outcome measures in adults with moderate-to-severe asthma. J Allergy Clin Immunol Pract. (2023) 11:3680–3689.e7. doi: 10.1016/j.jaip.2023.08.032 37652347

[18] Vissers LET Waller MA van der Schouw YT Hebert JR Shivappa N Schoenaker DAJM. The relationship between the dietary inflammatory index and risk of total cardiovascular disease, ischemic heart disease and cerebrovascular disease: findings from an Australian population-based prospective cohort study of women. Atherosclerosis. (2016) 253:164–70. doi: 10.1016/j.atherosclerosis.2016.07.929 27498398

[19] Hariharan R Odjidja EN Scott D Shivappa N Hébert JR Hodge A. The dietary inflammatory index, obesity, type 2 diabetes, and cardiovascular risk factors and diseases. Obes Rev. (2022) 23:e13349. doi: 10.1111/obr.13349 34708499

[20] Zhao S Gao W Li J Sun M Fang J Tong L. Dietary inflammatory index and osteoporosis: the National Health and nutrition examination survey, 2017–2018. Endocrine. (2022) 78:587–96. doi: 10.1007/s12020-022-03178-6 36044108

[21] Zhao W-H Huang Z-P Zhang X He L Willett W Wang J-L. Reproducibility and validity of a Chinese food frequency questionnaire. Biomed Environ Sci. (2010) 23:1–38. doi: 10.1016/S0895-3988(11)60014-720486429

[22] Huang Q Zhou X Zhang C Huang L Wang Q Chen Q. Relative validity and reproducibility of dietary measurements assessed by a Semiquantitative food frequency questionnaire among Chinese healthy adults. Nutrients. (2023) 15:545. doi: 10.3390/nu15030545 36771253 PMC9920913

[23] Yuexin Y Guangya W Xingchang P. China food composition. Beijing: Peking University Medical Press (2009).

[24] Hébert JR Shivappa N Wirth MD Hussey JR Hurley TG. Perspective: the dietary inflammatory index (DII)-lessons learned, improvements made, and future directions. Adv Nutr. (2019) 10:185–95. doi: 10.1093/advances/nmy071 30615051 PMC6416047

[25] Zhang Y Wu Y Zhang Y Cao D He H Cao X. Dietary inflammatory index, and depression and mortality risk associations in U.S. adults, with a special focus on cancer survivors. Front Nutr. (2022) 9:1034323. doi: 10.3389/fnut.2022.1034323 36590206 PMC9795013

[26] von Elm E Altman DG Egger M Pocock SJ Gøtzsche PC Vandenbroucke JP. The strengthening the reporting of observational studies in epidemiology (STROBE) statement: guidelines for reporting observational studies. J Clin Epidemiol. (2008) 61:344–9. doi: 10.1016/j.jclinepi.2007.11.008 18313558

[27] Han Y-Y Forno E Shivappa N Wirth MD Hébert JR Celedón JC. The dietary inflammatory index and current wheeze among children and adults in the United States. J Allergy Clin Immunol Pract. (2018) 6:834–841.e2. doi: 10.1016/j.jaip.2017.12.029 29426751 PMC5948124

[28] Schütte O Bachmann L Shivappa N Hebert JR Felix JF Röder S. Pro-inflammatory diet pictured in children with atopic dermatitis or food allergy: nutritional data of the LiNA cohort. Front Nutr. (2022) 9:868872. doi: 10.3389/fnut.2022.868872 35464023 PMC9024336

[29] Oh HY Lee S-Y Yoon J Cho H-J Kim Y-H Suh DI. Vegetable dietary pattern may protect mild and persistent allergic rhinitis phenotype depending on genetic risk in school children. Pediatr Allergy Immunol. (2020) 31:920–9. doi: 10.1111/pai.13308 32524629

[30] Lin Y-P Kao Y-C Pan W-H Yang Y-H Chen Y-C Lee YL. Associations between respiratory diseases and dietary patterns derived by factor analysis and reduced Rank regression. Ann Nutr Metab. (2016) 68:306–14. doi: 10.1159/000447367 27347884

[31] Kim SY Sim S Park B Kim J-H Choi HG. High-fat and low-carbohydrate diets are associated with allergic rhinitis but not asthma or atopic dermatitis in children. PLoS One. (2016) 11:e0150202. doi: 10.1371/journal.pone.0150202 26919190 PMC4769275

[32] Dyall SC Balas L Bazan NG Brenna JT Chiang N da Costa SF. Polyunsaturated fatty acids and fatty acid-derived lipid mediators: recent advances in the understanding of their biosynthesis, structures, and functions. Prog Lipid Res. (2022) 86:101165. doi: 10.1016/j.plipres.2022.101165 35508275 PMC9346631

[33] Ding Y Wang Y Zhang Y Dang B Hu S Zhao C. Alpha-linolenic acid improves nasal mucosa epithelial barrier function in allergic rhinitis by arresting CD4+ T cell differentiation via IL-4Rα-JAK2-STAT3 pathway. Phytomedicine. (2023) 116:154825. doi: 10.1016/j.phymed.2023.154825 37178572

[34] Sawane K Nagatake T Hosomi K Hirata S-I Adachi J Abe Y. Dietary Omega-3 fatty acid dampens allergic rhinitis via eosinophilic production of the anti-allergic lipid mediator 15-Hydroxyeicosapentaenoic acid in mice. Nutrients. (2019) 11:2868. doi: 10.3390/nu11122868 31766714 PMC6950470

[35] Magnusson J Kull I Westman M Håkansson N Wolk A Melén E. Fish and polyunsaturated fat intake and development of allergic and nonallergic rhinitis. J Allergy Clin Immunol. (2015) 136:1247–2. doi: 10.1016/j.jaci.2015.05.030 26152316

[36] Bojková B Winklewski PJ Wszedybyl-Winklewska M. Dietary fat and Cancer-which is good, which is bad, and the body of evidence. Int J Mol Sci. (2020) 21:4114. doi: 10.3390/ijms21114114 32526973 PMC7312362

[37] Kompauer I Heinrich J Wolfram G Linseisen J. Association of carotenoids, tocopherols and vitamin C in plasma with allergic rhinitis and allergic sensitisation in adults. Public Health Nutr. (2006) 9:472–9. doi: 10.1079/PHN2005868 16870019

[38] Wen W Yang F Shen X Feng N Ha H Ma R. Regulatory role of zinc in allergic rhinitis through the IL-33/ST2 pathway. J Healthc Eng. (2022) 2022:1–6. doi: 10.1155/2022/3718317 PMC8759868 35035826

[39] Petje L-M Jensen SA Szikora S Sulzbacher M Bartosik T Pjevac P. Functional iron-deficiency in women with allergic rhinitis is associated with symptoms after nasal provocation and lack of iron-sequestering microbes. Allergy. (2021) 76:2882–6. doi: 10.1111/all.14960 34037999 PMC8453563

[40] Li Q Zhou Q Zhang G Tian X Li Y Wang Z. Vitamin D supplementation and allergic diseases during childhood: a systematic review and Meta-analysis. Nutrients. (2022) 14:3947. doi: 10.3390/nu14193947 36235600 PMC9571357

[41] Jiang J Mehrabi Nasab E Athari SM Athari SS. Effects of vitamin E and selenium on allergic rhinitis and asthma pathophysiology. Respir Physiol Neurobiol. (2021) 286:103614. doi: 10.1016/j.resp.2020.103614 33422684

[42] Lee H Lee K Son S Kim Y-C Kwak JW Kim HG. Association of allergic diseases and related conditions with dietary fiber intake in Korean adults. Int J Environ Res Public Health. (2021) 18:2889. doi: 10.3390/ijerph18062889 33808963 PMC7998737

[43] Hosseini SA Shateri Z Abolnezhadian F Maraghi E Haddadzadeh Shoushtari M Zilaee M. Does pomegranate extract supplementation improve the clinical symptoms of patients with allergic asthma? A double-blind, randomized, placebo-controlled trial. Front Pharmacol. (2023) 14:1109966. doi: 10.3389/fphar.2023.1109966 36762119 PMC9905411

[44] Guasch-Ferré M Willett WC. The Mediterranean diet and health: a comprehensive overview. J Intern Med. (2021) 290:549–66. doi: 10.1111/joim.13333 34423871

[45] Chatzi L Apostolaki G Bibakis I Skypala I Bibaki-Liakou V Tzanakis N. Protective effect of fruits, vegetables and the Mediterranean diet on asthma and allergies among children in Crete. Thorax. (2007) 62:677–83. doi: 10.1136/thx.2006.069419 17412780 PMC2117278

[46] Tamay Z Akcay A Ergin A Guler N. Effects of dietary habits and risk factors on allergic rhinitis prevalence among Turkish adolescents. Int J Pediatr Otorhinolaryngol. (2013) 77:1416–23. doi: 10.1016/j.ijporl.2013.05.014 23820188

[47] Clark JS Dyer KA Davis CR Shivappa N Hébert JR Woodman R. Adherence to a Mediterranean diet for 6 months improves the dietary inflammatory index in a Western population: results from the MedLey study. Nutrients. (2023) 15:366. doi: 10.3390/nu15020366 36678237 PMC9866776

[48] Eifan AO Durham SR. Pathogenesis of rhinitis. Clin Exp Allergy. (2016) 46:1139–51. doi: 10.1111/cea.12780 27434218

[49] Peng Y-Q Wu Z-C Xu Z-B Fang S-B Chen D-H Zhang H-Y. Mesenchymal stromal cells-derived small extracellular vesicles modulate DC function to suppress Th2 responses via IL-10 in patients with allergic rhinitis. Eur J Immunol. (2022) 52:1129–40. doi: 10.1002/eji.202149497 35415925 PMC9545324

[50] Golebski K Layhadi JA Sahiner U Steveling-Klein EH Lenormand MM Li RCY. Induction of IL-10-producing type 2 innate lymphoid cells by allergen immunotherapy is associated with clinical response. Immunity. (2021) 54:291–307.e7. doi: 10.1016/j.immuni.2020.12.013 33450188

[51] Yalcin AD Gumuslu S Parlak GE Bisgin A Yildiz M Kargi A. Systemic levels of ceruloplasmin oxidase activity in allergic asthma and allergic rhinitis. Immunopharmacol Immunotoxicol. (2012) 34:1047–53. doi: 10.3109/08923973.2012.697902 22737977

